# Body composition estimation from selected slices: equations computed from a new semi-automatic thresholding method developed on whole-body CT scans

**DOI:** 10.7717/peerj.3302

**Published:** 2017-05-18

**Authors:** Alizé Lacoste Jeanson, Ján Dupej, Chiara Villa, Jaroslav Brůžek

**Affiliations:** 1 Faculty of Natural Sciences, Department of Anthropology and Human Genetics, Charles University, Prague, Czech Republic; 2 Faculty of Mathematics and Physics, Department of Software and Computer Science Education, Charles University, Prague, Czech Republic; 3 Department of Forensic Medicine, Unit of Forensic Anthropology, University of Copenhagen, Copenhagen, Denmark; 4 PACEA, UMR 5199, CNRS, Université de Bordeaux, Bordeaux, France

**Keywords:** Whole-body CT scans, Body composition, Single slices, Lean tissue, Adipose tissue, Volume and mass estimation

## Abstract

**Background:**

Estimating volumes and masses of total body components is important for the study and treatment monitoring of nutrition and nutrition-related disorders, cancer, joint replacement, energy-expenditure and exercise physiology. While several equations have been offered for estimating total body components from MRI slices, no reliable and tested method exists for CT scans. For the first time, body composition data was derived from 41 high-resolution whole-body CT scans. From these data, we defined equations for estimating volumes and masses of total body AT and LT from corresponding tissue areas measured in selected CT scan slices.

**Methods:**

We present a new semi-automatic approach to defining the density cutoff between adipose tissue (AT) and lean tissue (LT) in such material. An intra-class correlation coefficient (ICC) was used to validate the method. The equations for estimating the whole-body composition volume and mass from areas measured in selected slices were modeled with ordinary least squares (OLS) linear regressions and support vector machine regression (SVMR).

**Results and Discussion:**

The best predictive equation for total body AT volume was based on the AT area of a single slice located between the 4th and 5th lumbar vertebrae (L4-L5) and produced lower prediction errors (|PE| = 1.86 liters, %PE = 8.77) than previous equations also based on CT scans. The LT area of the mid-thigh provided the lowest prediction errors (|PE| = 2.52 liters, %PE = 7.08) for estimating whole-body LT volume. We also present equations to predict total body AT and LT masses from a slice located at L4-L5 that resulted in reduced error compared with the previously published equations based on CT scans. The multislice SVMR predictor gave the theoretical upper limit for prediction precision of volumes and cross-validated the results.

## Introduction

Body composition analysis is a vast field of research that encompasses a wide range of analytical procedures. This diversity is reflected in the existence of different levels of body composition, i.e., atomic, molecular, cellular, tissue system. At the tissue system level, body components that can be distinguished include the following (Mourtzakis et al., 2008; Heymsfield, 2008): bone, skeletal muscle, visceral organs, brain, and adipose tissue (AT). Total AT can be divided into the following anatomic groupings (Shen et al., 2003): bone marrow, subcutaneous AT and internal AT (that is both inter-visceral and inter-muscular). In the present paper, we refer to bone, skeletal muscle, visceral organs and the brain grouped together as total lean tissue (LT) in contrast to total AT (Kvist et al., 1990).

Readily available, techniques such as dual-energy X-ray absorptiometry (DXA) and bioelectrical impedance analysis (BIA) are widely used to estimate body composition. Being fast, noninvasive and not expensive, DXA is usually seen as the gold standard for estimating body composition. Estimating the volumes and masses of total body components, i.e., either AT or LT, is paramount in numerous areas, such as in research on nutrition and nutrition-related disorders like obesity, sarcopenia and anorexia nervosa (Ballor & Katch, 1989; Duren et al., 2008; Bredella et al., 2010; Thibault, Genton & Pichard, 2012; Prado & Heymsfield, 2014; Pedrera-Zamorano et al., 2014; Lee et al., 2015); cancer treatment and joint-replacement, in which knowing AT and LT proportions is important for the planning of treatment or surgery and for following the patient’s response to treatment or surgery as well as the evolution of the disease (Mourtzakis et al., 2008; Prado, 2013; Prado & Heymsfield, 2014); and research into energy-expenditure and exercise physiology (Wells, 2012; Mazzoccoli, 2016). DXA scanners are not always available in all those clinical settings (Mourtzakis et al., 2008) but lower limb CT scans in particular are often performed in these medical contexts. Therefore, being able to estimate body composition from CT scans would benefit several clinical areas without requiring additional irradiation or examinations.

Computed tomography (CT) and magnetic resonance imaging (MRI) offer direct images of the body components that can be measured straightforward in an accurate and reliable manner (Rössner et al., 1990; Leahy, 2011; Shuster et al., 2012). Moreover, it has been demonstrated that body components measured on specific CT scan slices are highly correlated with masses of whole-body tissues as estimated by DXA (Mourtzakis et al., 2008) and volumes estimates either based on 22 CT slices (Kvist et al., 1988a) or on approximately 40 MRI axial images (Shen et al., 2004b). Several equations have been derived from these data for the utilization of single slices at the waist level as surrogates for estimating total body composition. Although very useful in a clinical context, the most recent equations (derived from DXA body composition data) have been demonstrated to be inaccurate in estimating total body components volumes (Kilgour et al., 2016). However, high-resolution whole-body CT scans have not thus far been used to determine total body composition more accurately than DXA, nor have equations for estimating total body AT and LT from single slices been derived from such data.

Therefore, the first aim of this study was to determine a method to directly measure whole-body components data from whole-body CT scans. The second objective was to use this data to improve the existing equations for estimating total body AT and LT volumes and masses from selected CT scan slices. First, we introduce a new approach to measure body composition from whole-body CT scans data and we present its validation test. Then, we present new equations derived from this data for estimating body composition based on measurements of tissue areas on selected CT scan slices.

## Materials

We analyzed part of a dataset of whole-body CT scans performed on cadavers at the University of Copenhagen, Department of Forensic Medicine, Unit of Forensic Anthropology in Copenhagen, Denmark (Villa et al., 2017). No formal ethical consent is needed from Danish ethical committees to work with CT images of dead humans. Medico-legal autopsies are mandated by the police and CT scans are part of the routine investigation at the Department of Forensic Medicine (University of Copenhagen). The Department of Forensic Medicine adheres to Danish standards accreditation regarding data security. No personalized data can be exported from the systems. All personal data are removed from all images, as they have no use in the project; only age, sex, weight and height data were retained. Bodies were scanned within three days after death and exhibited very limited or none sign of decomposition The bodies were stored in a cold environment of 4–5 °C prior to being scanned. The sample was randomly constituted; it corresponds to people who died when the data was collected (Villa et al., 2017). The sample consisted of 36 males and five females with a range of weight that encompasses a large diversity (Table 1); five individuals are considered underweight, 20 have a weight within the physiological range, 15 are considered overweight and one is considered obese. The assignation of nutritional statuses corresponds to the BMI classification established by the WHO in 1995 (World Health Organization, 1995).

**Table 1:** Sample characteristics.

	♀ (*n* = 5)			♂ (*n* = 36)			♀+♂ (*n* = 41)		
	Mean	SD	Range	Mean	SD	Range	Mean	SD	Range
Age (years)	55	9	48–71	53	14.1	20–87	53.3	13.5	20–81
Body weight (kg)	51	13.1	40–73	70.9	13.2	46–98	68.4	14.6	40–98
Stature (cm)	161	3.5	158–166	173.2	6.8	161–188	171.7	7.6	158–188

**Notes.**

SD is the standard deviation.

Bodies have been weighed on an electronic scale with clothes removed prior to autopsy. Light devices such as intra-uterine devices and tubes from operations were left in place. The stature was measured using a metal ruler from the sole to the top of the head on corpses that were lying in a horizontal position. Males have been scanned using a Siemens Sensation 4 scanner with the following settings: 120 kV, 112.50 mAs, 2 or 3 mm slice thickness, 2 or 3 mm increment and either B30f, B31f or B60f reconstruction algorithms. Male stacks were combined from one (*n* = 5), two (*n* = 34) or three (*n* = 2) partial scans. Female scans were entirely performed in one run, using a Siemens Somatom Definition CT scanner with the following settings: 120 kV, 190 mAs, 3 mm slice thickness, 3 mm increment and a B30f reconstruction algorithm.

## Methods

### Body composition assessment on whole-body CT scans

We used Mimics^®^ (Materialise Interactive Medical Image Control System, Materialise HQ, Leuven, Belgium) for the majority of the analysis. Mimics^®^ is an image processing software for 3D design and modeling. The freeware FIJI (Schindelin et al., 2012; Schneider, Rasband & Eliceiri, 2012) was used to identify the optimal AT density range.

In order to assess body composition from whole-body CT scans, issues regarding the distinction between AT and LT densities across the whole body had to be addressed. Techniques usually used to distinguish AT from the other body components involve either manual correction, encircling zones, the use of fixed density ranges, selection of a region of interest based on edge-detection filters or watersheds or combinations of these techniques (Kvist et al., 1988b; Mitsiopoulos et al., 1998; Lemieux, Lesage & Bergeron, 1999; Yoshizumi, Nakamura & Yamane, 1999; Irving et al., 2007; Mourtzakis et al., 2008; Bredella et al., 2010). These techniques are not applicable to whole-body CT scans, given the large number of images that compose a whole-body scan stack, the need of determining intra- or peri-muscular ATs that are not regularly located, and the existence of a large number of structures that constitute the total body AT (Shen et al., 2003). Moreover, the density ranges for AT vary; the thresholds are typically located between −190 and −30 Hounsfield units (HU) for subcutaneous AT and between −250 and −50 for visceral AT. A study (Rössner et al., 1990) revealed similar AT areas at the waist level based on measurements from cross-sectional planimetry of two cadavers and measurements from CT scans slices of the same individuals in which the AT was defined from −250 to −50, or −190 to −30 or −140 to −40. However, while the differences based on single slices taken at the waist level are not significant, the different ranges of HUs used for the definition of AT on the whole-body CT scans created substantial discrepancies in our sample. There are also advanced segmentation methods (Zhang & Wu, 2011), however they are generally not implemented in softwares used for clinical purposes.

Given these limitations, we established a repeatable protocol for differentiating AT and LT densities on whole-body CT scans that is calibrated on an individual basis to mitigate the errors introduced by different scan parameters.

**Figure 1:**
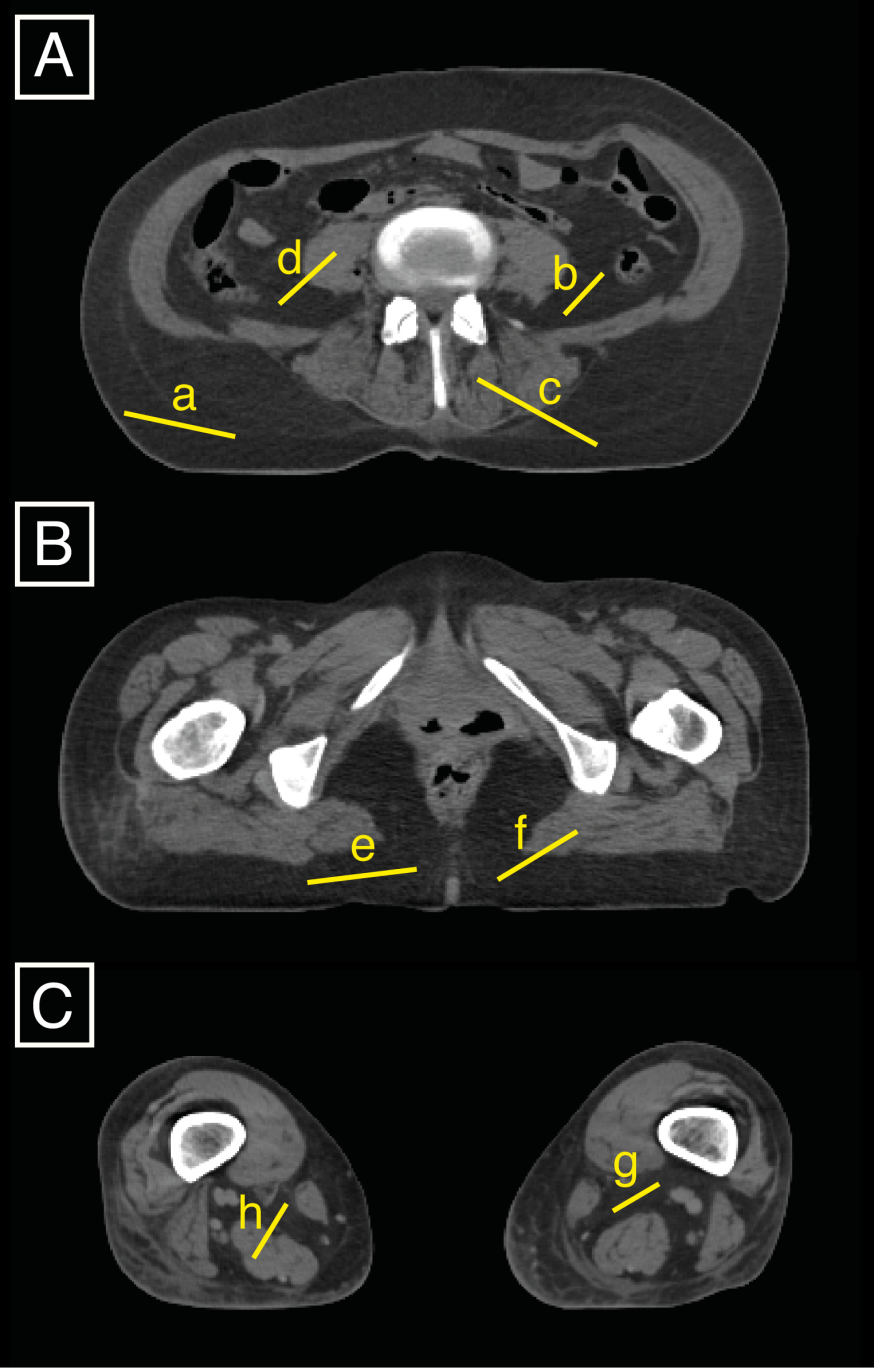
Three slices (A is between the 4th and 5th lumbar vertebrae, B is at 85% of the maximum femoral length, and C is at 15% of the maximum femoral length) were used to set the AT upper density limit by averaging eight specific upper density limits that can be displayed in FIJI by drawing lines (a to h) on the slices and displaying the related histograms.

We set the lower AT threshold to -205 HU, which is the default setting in Mimics^®^. To determine the AT density upper limit (and cutoff with LT), we defined a strategy for whole-body CT scans developing the protocols of Kvist et al. (1990) and Lemieux, Lesage & Bergeron (1999). We averaged the upper density limits on a total of three slices at eight locations, including four AT upper density limits and four upper density limits of the transition between AT and LT by using the tool to plot histograms from drawn lines in FIJI (Fig. 1). The three selected slices corresponded to well-defined anatomical locations, i.e., the waist (between the 4th and 5th lumbar vertebrae), the gluteal region (defined in this study as being located at 85% of maximum femoral length) and the knee (defined in the present study as being located at 15% of the maximum femoral length). The densities of these eight locations were displayed one by one on eight histograms in FIJI and corresponded to three different types of AT: visceral AT, muscular AT, and subcutaneous AT. The latter, being the largest amount of AT existing in the body, was plotted twice (both at the waist and buttock levels) which reduces the unnecessary inter-rater variation. To get the eight upper densities that we then averaged and took as the cutoff density between AT and LT, we proceed such as follows:

• On a slice located between the 4th and 5th lumbar vertebrae (subsequently called L4-L5 level), four histograms are generated in FIJI (using the shortcut cmd+K after drawing one line at a time) as illustrated in Fig. 1A: lines (a) and (b) correspond to 100% of the subcutaneous AT and visceral AT, respectively; and lines (c) and (d) are traced to obtain histograms of approximately 50% of the LT (either erector spinae or transversospinal muscles or kidney) and 50% of the subcutaneous AT and visceral AT, respectively.
• At 85% of the maximum femoral length (Fig. 1B), two density histograms are generated. One histogram, generated from line (e), is composed of 100% of the subcutaneous AT, and the other histogram, from line (f), represents the density of the line that passes from the muscle (gluteus maximus) to the subcutaneous AT with the middle of the line located at the transition between the two tissues.
• The slice located at 15% of the maximum femoral length (Fig. 1C) is used to draw two lines that are composed of 100% of the muscular AT (g) and contain approximately 50% of the muscle and 50% of the muscular ATs (h), respectively. The knee level was chosen because it conveniently and quite clearly displays the muscular AT.

In our sample, the AT upper density limit was typically found between −16 and 26 HU (mean = −3, SD = 10). Once the upper density limit for the AT was found, the LT inferior density limit was set to correspond to the AT upper density limit +1 HU. The superior density limit corresponds to the maximum attenuation unit (in HU) of the scan.

Prior to voxel classification, we denoised the whole stack of images using a median filter, with a neighborhood size of 3 × 3 × 3 voxels. This filter suppresses noise, but does not distort edges.

**Figure 2:**
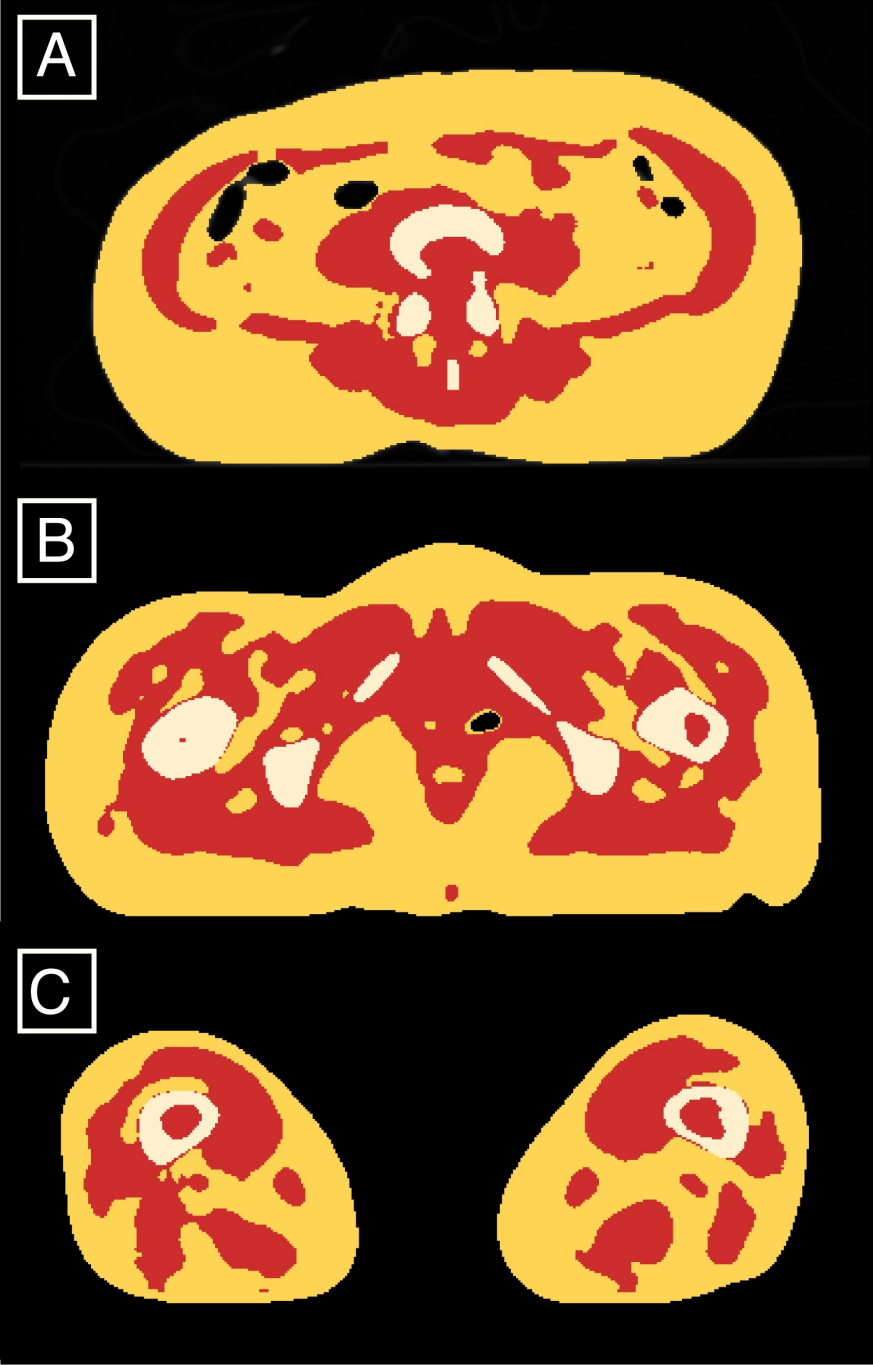
Classified AT (red) and LT (yellow and white) exemplified on the slices located between the 4th and 5th lumbar vertebrae (A), at 85% of the maximum femoral length (B) and at 15% of the maximum femoral length (C).

The processing of CT scans involves the creation of masks that correspond to the body components (AT and LT). Following the protocol described above for tissue densities determination in whole-body CT scans, we were able to display masks corresponding to the AT and LT volumes for the whole body (exemplified for specific slices on Fig. 2). The mask volumes were recorded in liters for the total body and in cm^2^ for the three single-slices located between the 3rd and 4th lumbar vertebrae (L3-L4), L4-L5 and at the mid-thigh (halfway through the maximum femoral length).

**Figure 3:**
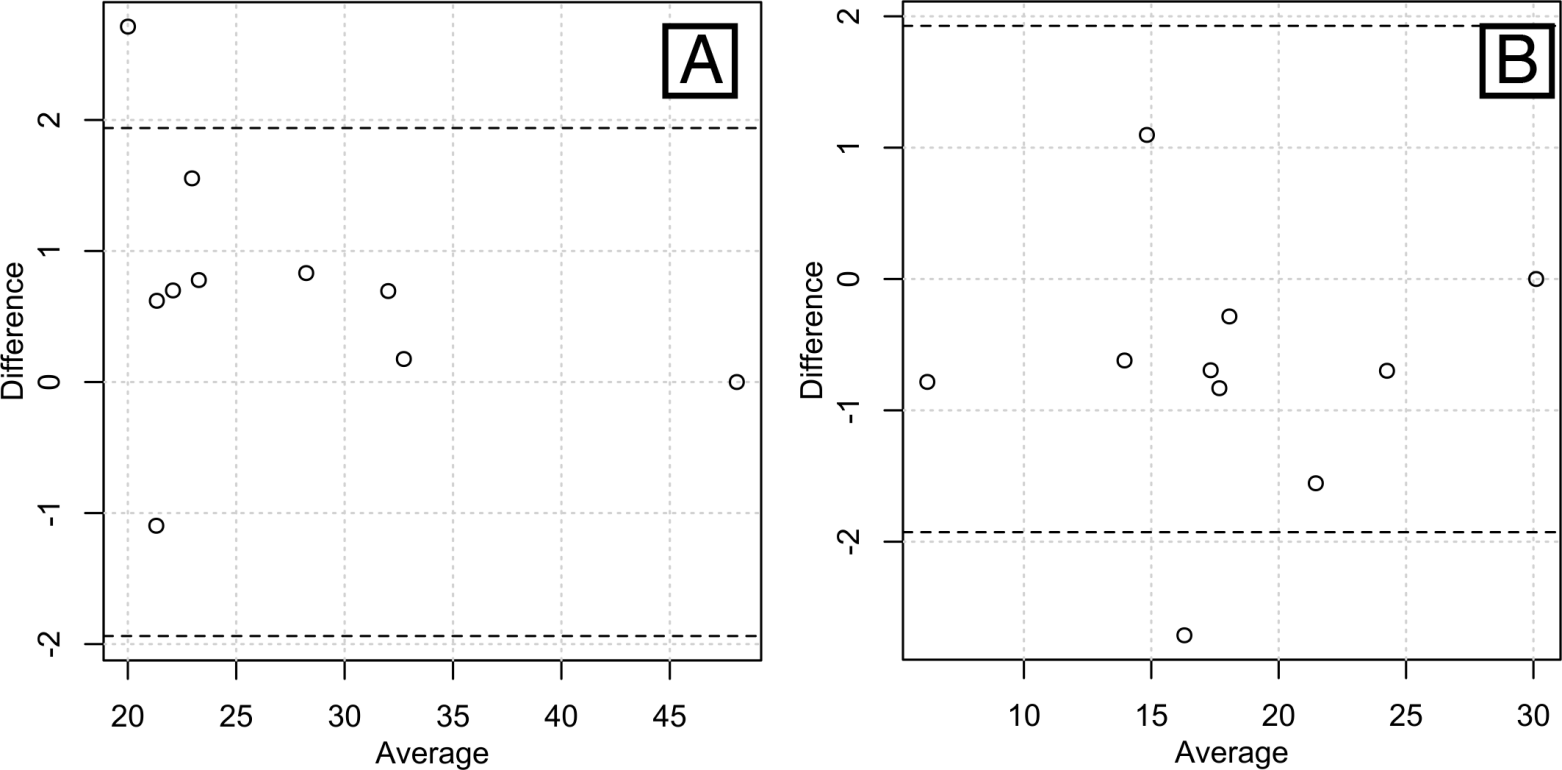
Bland-Altman plots of the inter-rater agreements in measuring total lean volume (A) and total fat volume (B). Most of the pairs of data are within the 95% confidence intervals (bounded by the dashed lines), showing a high inter-rater agreement.

We evaluated the repeatability of our tissue segmentation protocol using intra-class correlation coefficients (ICCs) (Landis & Koch, 1977) and Bland-Altman plots (Fig. 3). Ten different scans (from five males and five females encompassing a large range of BMIs, i.e., one underweight, three normal-range and one overweight subject for each sex) were processed. The resulting AT and LT volumes were used to calculate the ICCs. To evaluate the inter-rater error, the first author acted as the first rater (ALJ) and the second rater was a non-trained intern in bio-anthropology. He received a written protocol similar to the one described above along with rapid training. The intra-rater error was assessed based on the performance of the first observer in rating the same sub-sample twice at an interval of two weeks. We obtained ICCs of 0.9803 for the AT and 0.8831 for the LT between raters. ICCs of 0.9833 for the AT and 0.9909 for the LT were obtained for the intra-rater agreement. These findings are consistent with nearly perfect agreement between the raters. Additionally, the B-A plots for the AT and LT volumes obtained from the two raters show that most pairs of points display a difference of ±1 liter and almost all of them are situated within the 95% confidence intervals (dashed lines on Fig. 3).

The total body AT and LT volumes obtained from our sample are provided in Table 2. Additionally, we calculated the AT and LT masses (Table 2). The AT masses were converted from liters using the known density of fat, i.e., 0.92 kg per liter (Kvist et al., 1990). The LT masses were obtained by the subtraction of the AT masses from known weights.

**Table 2:** Whole-body AT and LT volume (in mm^3^) and mass (in kg) data of the sex-pooled sample (*n* = 41).

	Volume			Mass		
	Mean	SD	Range	Mean	SD	Range
Adipose tissue	22.7	9.4	5.8; 43.3	20.9	8.6	5.3; 39.8
Lean tissue	40	9.3	18.4; 58	47.5	9.4	31.2; 64.7

**Notes.**

SD is the standard deviation.

### Body composition estimation from selected slices

We used ordinary least squares (OLS) linear regression to identify linear equations that related the total body tissue volumes and masses to the corresponding areas from the specific slices. In all cases, we checked the assumptions of the OLS regressions. These tests included Durbin-Watson test for residual autocorrelation and the Breusch-Pagan test for residual heteroscedasticity. The residual normality was checked using a Q-Q plot, and finally, ANOVA was performed to verify the significance of the linear fit. We evaluated these fits and those of the two other sets of equations published for body composition prediction from single CT scan slices (Kvist et al., 1988a; Mourtzakis et al., 2008) using the mean absolute error of prediction (|PE|). This measure is simply the average of the absolute values taken from the differences between the measured and predicted values. We also compared the results with the mean percent prediction error (%PE) calculated as ((observed − predicted)∕predicted)∗100. We compared the accuracies of the combined and separate equations in terms of the mean absolute error using a paired Wilcoxon signed-rank test.

Finally, we used support vector machine regression (SVMR) to create a prediction model of AT and LT volumes based on AT and LT areas in all of the L3-L4, L4-L5 and mid-thigh slices. A linear kernel was used. The model was fit to the entire sample without using sex as a factor. Again, the accuracy of prediction was evaluated with the mean absolute error. Furthermore, we checked for over fitting by calculating these mean absolute errors with leave-one-out cross validation.

All statistical processing was performed with R (R Development Core Team, 2015), using the packages irr (Gamer et al., 2005) and e1071 (Meyer et al., 2015). In all cases, statistical significance was defined at the level of α = 0.05.

## Results

### Total body AT volume

Although it has been done several times from MRI data, Kvist et al.’s study (Kvist et al., 1988a) is, as far as we know, the only one that aimed to set an estimate of total body composition from CT scans slices. Kvist et al.’s equations have been set up to estimate total body composition from a single-slice located at L4-L5 level. The regressions are based on a set of 22 slices that were used to derive whole-body composition data. AT was quantified from toe to finger tips (arms stretched over the head) by encircling the areas of interest with a light pen and calculating the pixel distribution with attenuation values between −190 and −30 HU on 22 slices. Knowing the distances between each scan, the volume was derived according to the equation $V={\mathop{\sum }\nolimits }_{i=1}^{23} \frac{{a}_{i} \left( {b}_{i}+{c}_{i} \right) }{2} $ where *a*_*j*_ is the distance between scans and *b*_*j*_ and *c*_*j*_ are the AT areas in adjacent scans. The equations are sex-specific and based on a sample that encompasses quite a wide range of body sizes.

**Figure 4:**
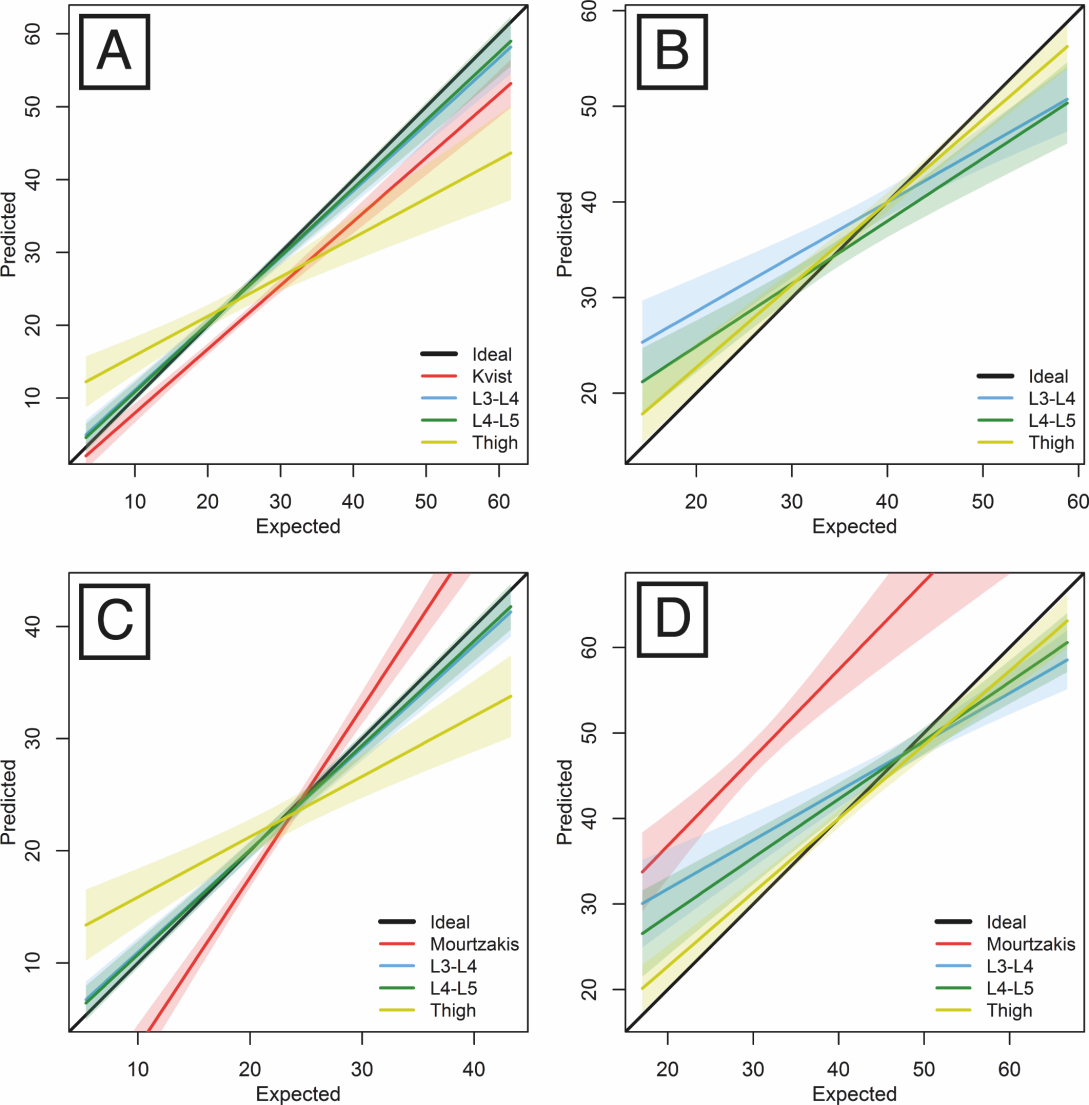
Linear regression predicting whole-body AT volume (A), LT volume (B), AT mass (C), LT mass (D) by respective areas measured on singles-slices located at either L3-L4, L4-L5 or thigh levels.

Using the total body AT data derived from the whole-body CT scans in our sample of 41 individuals, we were able to test Kvist et al.’s predictive equation against our new predictive equations based on the AT areas at the mid-thigh, L3-L4 and L4-L5 levels and evaluate their respective mean errors (Table 3 and Fig. 4A).

The error was lower when the sex-specific equations were applied in our sample, but this difference was not significant. Moreover, all of the ANOVA *p*-values were below 0.0001. We therefore present only the equations for the sex-combined samples. The mean errors were lower for the new regressions than for Kvist et al.’s sex-specific regressions despite the new regressions being sex-pooled. Kvist et al. did not provide any insight into the sex-specificity, although neither the results nor the errors seem to differ significantly between the sexes. Moreover, our sample contained only 5 females, which is clearly insufficient for defining separate equations.

**Table 3:** Predictive equations and their respective mean errors in the estimation of the whole-body AT volume (liters) from the AT areas (cm^2^) of single CT scan slices.

Study	Models	Equations	|PE|	%PE
Kvist et al. (1988a)	♀ whole-body AT volume estimated from AT area at L4-L5 level	0.0778∗*AT*_*L*4-*L*5_ − 0.59	2.27	18.55
	♂ whole-body AT volume estimated from AT area at L4-L5 level	0.0693∗*AT*_*L*4-*L*5_ + 0.09	4	17.55
Current study	♀+♂ whole-body AT volume estimated from AT area at L3-L4 level	0.069∗*AT*_*L*3-*L*4_ + 4.691	2.20	11.56
	♀+♂ whole-body AT volume estimated from AT area at L4-L5 level	0.074∗*AT*_*L*4-*L*5_ + 2.737	1.86	8.77
	♀+♂ whole-body AT volume from AT area at mid-thigh level	0.173∗*AT*_*mid*-*thigh*_ + 5.543	5.09	24.06

**Notes.**

|PE| is the mean absolute prediction error, provided in liters. %PE is the mean percent prediction error calculated as ((observed − predicted)∕predicted)∗100.

### Total body LT volume

We also tested the performance of the equations in the estimation of the whole-body LT volumes from single slices (Table 4 and Fig. 4B). The whole-body LT volume estimates produced the least error when based on the mid-thigh slice (in comparison with either waist slice).

### Total body AT and LT masses

One study (Mourtzakis et al., 2008) has provided a set of equations for predicting the total body fat (FM) and fat-free masses (FFM) from a AT and LT areas of two CT scan slices located between the 3rd and 4th lumbar vertebrae. The AT and LT areas were determined with fixed density ranges (manually corrected when needed) of −190 to −30 HU and −29 to 150 HU, respectively. The FM and FFM data were derived from DXA analysis.

Using the total body AT and LT mass data derived from our sample of whole-body CT scans, we have provided a set of equations for estimating total body AT and LT masses from AT and LT areas measured on the slice that provides the lowest prediction error, i.e., the slice located at the L4-L5 level (Table 5 and Figs. 4C and 4D). A comparison of the results with those of Mourtzakis et al.’s sex-pooled equation estimates for our sample is also provided.

Similarly to the volume predictions, the prediction pattern for the whole-body tissue masses based on single slices exhibited lower mean absolute errors for the AT than for the LT. However, although the error for estimating masses was quite low, it was greater than the error for estimating volumes.

### Multislice predictions of total volumes

The multislice predictor based on SVM regression produced the most accurate predictions. The AT volume was predicted with a mean absolute error of 1.5 liters (1.93 liters with cross-validation). The LT volume was predicted with an error of 2.24 liters (2.35 liters cross-validated). The R script for the multislice predictor is provided in the Supplemental material.

**Table 4:** Sex-pooled predictive equations and their respective mean errors in the estimation of the whole-body LT volume (liters) from the LT area (cm^2^) of single CT scan slices.

Models	Equations	|PE|	%PE
♀+♂ whole-body LT volume estimated from LT area at L3-L4 level	0.147∗*LT*_*L*3-*L*4_ + 8.866	4.48	12.09
♀+♂ whole-body LT volume estimated from LT area at L4-L5 level	0.162∗*LT*_*L*4-*L*5_ + 5.586	3.63	12.42
♀+♂ whole-body LT volume estimated from LT area at mid-thigh level	0.108∗*LT*_*mid*-*thigh*_ + 13.021	2.52	7.08

**Notes.**

|PE| is the mean absolute prediction error, provided in liters. %PE is the mean percent prediction error calculated as ((observed − predicted)∕predicted)∗100.

**Table 5:** Predictive equations and their respective mean errors for the estimation of the whole-body AT and LT masses (kilograms) from the LT and AT areas (cm^2^) of single CT scan slices.

Study	Models	Equations	|PE|	%PE
Mourtzakis et al. (2008)	♀+♂ whole-body FM from AT area at L3-L4 level	0.042∗*AT*_*L*3-*L*4_ + 11.2	3.23	22.49
	♀+♂ whole-body FFM from LT area at L3-L4 level	0.14∗*LT*_*L*3-*L*4_ + 0.72	6.92	18.41
Current study	♀+♂ whole-body AT mass from AT area at L4-L5 level	0.0677∗*AT*_*L*4-*L*5_ + 2.5177	1.71	8.77
	♀+♂ whole-body LT mass from LT area at L4-L5 level	0.1839∗*LT*_*L*4-*L*5_ + 14.6903	3.67	8.01

**Notes.**

|PE| is the mean absolute prediction error, provided in kilograms. %PE is the mean percent prediction error calculated as ((observed − predicted)∕predicted)∗100.

## Discussion and Conclusions

First, the correspondence of real body composition versus body composition assessed through CT could be called into question. However, the volumes and masses of water-filled balloons and several organs (*in situ* and excised) have been demonstrated to be accurately measured in CT scans (Heymsfield et al., 1979). Similarly, AT surface measurements from CT slices and the corresponding cross-sectional planimetry at the waist level can provide very similar results for male cadavers (Rössner et al., 1990). As far as we know, the only study that actually assessed body composition from whole-body images is the one of Shen et al. (2004a) that is based on semiautomatic segmentation performed on an “in-house image segmentation software program” that is not readily available.

The correspondence of body composition measured on cold cadavers to body composition measured in the living could also be questioned. The only measured variables from CT scans in this study are adipose tissue and lean tissue. It has been shown that HU ranges of blood and serous fluids measured within a few days after death are comparable to those of the living (Zech et al., 2014). As for the influence of storage condition on radiodensities, the same experimental study demonstrated that mean HU ranges of the blood and serous fluid at temperatures of 4 to 40 °C only vary at a maximum of 10 HU and never overlap (Zech et al., 2014).

The protocol for assessing the statistical advantage of using sex-specific prediction equations over sex-pooled equations for the entire sample has been set up as follows. The prediction errors were calculated for each specimen (*n* = 41) with both the sex-separated and sex-pooled equations. These errors were tested using paired Wilcoxon signed-rank tests. Kvist et al.’s study (Kvist et al., 1988a) provided sex-specific equations but no specific justification was given. Mourtzakis et al.’s (2008) study assumed that sex did not influence the relationships between the areas of body components on a single slice and total body component masses based on a study (Shen et al., 2004a) that only addressed issues related to visceral adipose tissue and not any other tissue. These authors did not test the actual influence of sex on the results or equations. Nevertheless, even with our very limited sample of five females, it should be noted that the single-slices at the mid-femur location seemed to produce better, albeit non-significantly better, estimations of total body components in females than in males. Although this hypothesis still requires further testing on a larger sample, it correlates with previous assertions of a sex-dependent AT distribution that tends to be located more subcutaneously (as opposed to viscerally) and in the gluteal region in females up to a certain amount of total-body AT (Krotkiewski et al., 1983; Kvist et al., 1988a; Kvist et al., 1990; Després, 2012). Thus, to properly evaluate whether sex-specific equations provide significantly better results, we need to examine a dataset that includes more females.

Aging should not be a factor of influence on the reliability of our equations: the body composition variation between age categories is just a matter of differential amounts and proportions. Accordingly, variations in total body composition are reflected in the single slices used for prediction.

Body height is likely to have an influence on total body composition assessment, although this has not been evaluated in any other attempts to predict body composition from single slices (Kvist et al., 1988a; Mourtzakis et al., 2008). Body height would need to be tested as a regressor in further models. However, measuring slices located at L3-L4 or L4-L5 levels takes into account one of the maximum breadths of the body (corresponding to bicristal breadth) that is also one of the proxies for body size. It has been shown that this measurement is proportional to AT more than it is to LT (Chumlea et al., 2002) and this would explain why AT and LT areas at the waist level predict better AT and LT volumes and masses than those at the mid-thigh level.

The current study is an exploratory step into the definition of accurate and reliable equations for estimating total body AT and LT based on single slices and whole-body CT scan data. Due to the scarcity of available whole-body CT scans samples, the small size of our sample did not allow providing sex-separated equations, which would probably increase the accuracy of the equations. Although we checked the assumptions of the regressions to prevent for overfitting, the equations still need to be validated on an independent sample. It would also be interesting to compare how they perform in a wider sample of living humans CT scans with data of body composition estimated through DXA. It is however hypothesized that the results of body composition either estimated with DXA or CT scans would be very similar (Kvist et al., 1988a; Shen et al., 2004b; Mourtzakis et al., 2008).

We provide a sex-pooled equation for estimating the total body AT volume from a slice located at the L4-L5 level with a mean percent absolute error (8.94%) that is lower than those provided by the currently existing equations ([18.55; 18.59%]) in which the whole-body AT volume was derived from 22 CT slices (Kvist et al., 1988b). Additionally, we provided regressions to estimate the total body AT volumes from the L3-L4 and mid-thigh slices in addition to equations for estimating the total body LT volume from single slices. Similarly to Kvist et al.’s study (Kvist et al., 1988a), the best predictive equation for the total body AT volume was that based on the AT area at the L4-L5 level.

For the estimation of the LT volume, the best predictor was the LT area of the mid-thigh (%PE = 7.76). This result might be related to the fact that the mid-thigh slice displays a greater amount of LT than the L3-L4 and L5-L5 slices. Complementing our results, it has been shown (Marquis et al., 2002) that muscle area measured at mid-thigh is the best predictor of chronic obstructive pulmonary disease (COPD) mortality, as mid-thigh muscle area is an index of muscle mass which loss increases the risk of mortality in chronic diseases.

We also provide equations for estimating the total body AT and LT masses from a slice located at the L4-L5 level and compare them with the only available study based on CT scans. Our predictive equations provide lower mean percent prediction errors ([8.80; 8.92%] than those provided by Mourtzakis et al. (%PE = [18.41; 22.49]). Mourtzakis et al. (2008) provided a set of equations for predicting the total body fat (FM) and fat-free masses (FFM) from the AT and LT areas of two CT scan slices located between the 3rd and 4th lumbar vertebrae. Although Mourtzakis et al.’s equations have been found to be inaccurate by Kilgour et al. (2016), we chose to compare our equations against theirs because Mourtzakis et al.’s study is, to our knowledge, the only one that quantified the power of estimating body components masses from a single CT slice. It should be noted that DXA, which was used by Mourtzakis et al. to assess the body composition, provides data for fat mass (FM) and fat-free mass (FFM), which are different components than the AT and LT (Shen et al., 2003; Prado & Heymsfield, 2014). Adipose tissue is directly measurable through CT and MRI because it has a specific density; its chemical composition includes lipids (83%), water (2%), and proteins and minerals (2%). AT is subcutaneous but also visceral, interstitial (i.e., interspersed among the cells of the organs) and in the yellow marrow and has a density of 0.923 g/cm^3^ (Shen et al., 2003). In comparison, fat is indirectly measurable with DXA (via its subtraction from the fat-free mass or body cell mass). Chemically, fat is composed of lipids in the form of triglycerides. Fat is primarily found in the adipose tissue but also exists in other tissues and has a density of 0.900 g/cm^3^ (Shen et al., 2003). Although fat and AT are similar, they are not identical (Shen et al., 2003). Thus, the predictive power of our equations for estimating the AT and LT masses are comparable with those of Mourtzakis et al.’s equations only to a certain extent.

To further understand, it would be beneficial to measure skeletal muscle independently from LT, which would allow for the testing of Shen et al.’s (2004a) predictive equations based on single slices (located either 5 cm above or below L4-L5) and body composition data (skeletal muscle and adipose tissue, respectively) of whole-body MRIs.

Additionally, the prediction system based on the composition of multiple slices that we created produced the best predictions. This finding demonstrates that if the body components areas of several slices are known, these areas in conjunction with machine learning tools can be used to improve the estimations produced by single-slice methods. The LOOCV applied to the multi-slice predictions validated the results.

These results would not have been possible without a method for individually determining the AT densities on whole-body CT scans. This method was proven useful, reproducible, and reliable in the present study. While based on specific scanner parameters, this method is not dependent on the software used because it only employed a classical median denoising algorithm.

In summary, we assessed the total body AT and LT volumes and masses on whole-body CT scans of 41 people with a wide range of known weights. From this data, we tested two existing equations for estimating the total body AT volume from a single slice located between the 4th and 5th vertebrae (Kvist et al., 1988a) as well as two equations for estimating the AT and LT total body masses from a slice located at the L3-L4 level (Mourtzakis et al., 2008). The predictions have also been tested and cross-validated with the use of all three slices at once with SVMR, producing lower errors of prediction. The new equations produced lower prediction errors than the previously published equations and should be applicable to a broad range of weights and body composition.

##  Supplemental Information

10.7717/peerj.3302/supp-1 Data S1 Raw body composition data

10.7717/peerj.3302/supp-2 Supplemental Information 2 R script for predicting total body components volumes from multiple slices

## Additional Information and Declarations

### Data Availability

The following information was supplied regarding data availability: The raw data has been supplied as a Supplementary File.
