# Supplementary material for: Body composition estimation from selected slices: equations computed from a new semi-automatic thresholding method developed on whole-body CT scans
Source: PeerJ. 2017 May 18;5:e3302. doi: 10.7717/peerj.3302 (PMC5438582; doi:10.7717/peerj.3302)
Supplement: Data S1 [file peerj-05-3302-s001.pdf]

Body composition data

ID	Sex	fat-free_mm^3	fat-free_kg	fat_mm^3	fat_kg
1	M	43737749	55	37040245	34
2	M	36726150	40	30791006	28
3	M	48090107	54	30106455	28
4	M	33193757	38	19167104	18
5	M	41112051	45	10509184	10
6	M	32359462	34	16982979	16
7	M	41315658	43	19663180	18
8	M	33921891	40	31820155	29
9	M	46369251	52	23853983	22
10	M	28644806	32	17255737	16
11	M	40109301	41	15661682	14
12	M	29065607	32	22409664	21
13	M	32819530	34	17922186	17
14	M	46116489	54	18777020	17
15	M	35464795	38	29557635	27
16	M	25545691	29	43274253	40
17	M	42513124	47	10545945	10
18	M	30721487	32	23384648	22
19	M	30091049	29	17390666	16
20	M	39153185	44	35033020	32
21	M	44346110	49	23216545	21
22	M	43839132	47	37999983	35
23	M	35602902	38	24890983	23
24	M	35446161	40	18604641	17
25	M	33730370	39	35402954	33
26	M	31201728	33	25843735	24
27	M	35010643	39	25689886	24
28	M	51062576	39	39337013	36
29	M	35775337	44	23881866	22
30	M	14355844	27	20824106	19
31	M	20765558	23	15376049	14
32	M	35399331	41	8958741	8
33	M	42358095	47	17962719	17
34	M	40492756	42	10015505	9
35	M	25272886	30	12942400	12
36	M	42663133	49	40156426	37
37	F	23738220	28	20680014	19
38	F	21361750	25	14942534	14
39	F	21647257	26	13639062	13
40	F	23664270	29	5816743	5
41	F	22431115	44	23901718	22
