# Supplementary material for: Body composition estimation from selected slices: equations computed from a new semi-automatic thresholding method developed on whole-body CT scans
Source: PeerJ. 2017 May 18;5:e3302. doi: 10.7717/peerj.3302 (PMC5438582; doi:10.7717/peerj.3302)
Supplement: Supplemental Information 2 [file peerj-05-3302-s002.zip › Lacoste_Jeanson_et_al._multislice_predictor/ReadMe_multislice_predictor_total_BC.rtf]

Multislice predictor of whole-body lean tissue (LT) and adipose tissue (AT) volumesLacoste Jeanson A, Dupej J, Villa C, Brůek J 2017. Body composition estimation from selected slices: Equations computed from a new semi-automatic thresholding method developed on whole-body CT scans.	•	The script "bcpred.r" runs in R and needs the two .rds files to be in the same source directory.	•	For predicting whole-body LT volume from tissues areas measured on three CT scan slices, just type the following lines in R, replacing "a,b,c,d,e,f" by data of tissue areas in mm2 as	⁃a is AT area at L3-L4 level	⁃b is LT area at L3-L4 level	⁃c is AT area at L4-L5	⁃d is LT area at L4-L5	⁃e is AT at mid-thigh	⁃f e is LT mid-thigh	>	source("bcpred.r")	>	bcpred$lt.multi.alj(a,b,c,d,e,f)	•	For predicting whole-body AT volume from tissues areas measured on three CT scan slices, just type the following lines in R, replacing "a,b,c,d,e,f" by data of tissue areas in mm2 (see above)	>	source("bcpred.r")	>	bcpred$at.multi.alj(a,b,c,d,e,f)	•	The results are either LT or AT volumes in liters.
